# Cellular Plasticity of CD4+ T Cells in the Intestine

**DOI:** 10.3389/fimmu.2014.00488

**Published:** 2014-10-07

**Authors:** Verena Brucklacher-Waldert, Edward J. Carr, Michelle A. Linterman, Marc Veldhoen

**Affiliations:** ^1^Laboratory for Lymphocyte Signalling and Development, The Babraham Institute, Cambridge, UK

**Keywords:** T cells, plasticity, intestines, Th1 cells, Th17 cell

## Abstract

Barrier sites such as the gastrointestinal tract are in constant contact with the environment, which contains both beneficial and harmful components. The immune system at the epithelia must make the distinction between these components to balance tolerance, protection, and immunopathology. This is achieved via multifaceted immune recognition, highly organized lymphoid structures, and the interaction of many types of immune cells. The adaptive immune response in the gut is orchestrated by CD4^+^ helper T (Th) cells, which are integral to gut immunity. In recent years, it has become apparent that the functional identity of these Th cells is not as fixed as initially thought. Plasticity in differentiated T cell subsets has now been firmly established, in both health and disease. The gut, in particular, utilizes CD4^+^ T cell plasticity to mold CD4^+^ T cell phenotypes to maintain its finely poised balance of tolerance and inflammation and to encourage biodiversity within the enteric microbiome. In this review, we will discuss intestinal helper T cell plasticity and our current understanding of its mechanisms, including our growing knowledge of an evolutionarily ancient symbiosis between microbiota and malleable CD4^+^ T cell effectors.

## Introduction

The adult human gastrointestinal tract is the largest surface area of the body that contacts with the environment, covering 200–300 m^2^ (1). This intestinal surface is constantly exposed to a diverse range of foreign antigens originating from microorganisms (both commensals and pathogens) and food antigens from the diet (2). Commensal microorganisms play an essential role in extracting nutrients from food that are otherwise inaccessible to the host [such as the metabolism of vitamin K by *E. coli* (3)], they are required for the development of the host’s immune system and for the prevention of colonization of the gastrointestinal tract by pathogens. Mucosal pathogens, including viruses, fungi, parasites, and bacteria, can cause pathology either by local effects after mucosal colonization – such as inducing local inflammation or secreting toxins – or, through systemic infection after breeching mucosa. Microorganism-derived antigens, food-derived antigens, and airborne particles, can be potential immunogens. An inappropriate response to these immunogens at the mucosal surface can be detrimental, leading to local or systemic pathology that result in acute or chronic inflammation. Therefore, it is essential that the myriad of antigens present at the intestinal surface is dealt with appropriately to minimize potential danger and maximize host benefit. This protection is achieved by a flexible, multi-layered system of physical, and immunological barriers within the gastrointestinal tract.

A central part of the complex host defense system is gut-associated lymphoid tissue (GALT). GALT is a system of highly organized immune structures strategically placed along the entire gastrointestinal tract, containing specialized micro-environments where gut-derived antigens are presented by professional antigen presenting cells (APCs) to lymphocytes [reviewed recently in Ref. (4)]. The broad antigenic sampling within the GALT facilitates the interaction between rare antigen-specific B and T cells leading to the initiation of an appropriate adaptive immune response (5). CD4^+^ T cells are critical players in the adaptive immune response within the GALT. Naïve T cells egress from the thymus as immature T cells with a broad range of T cell receptors (TCRs) and can be activated in the periphery following encounter with their specific antigen. T cell activation is initiated by ligation of the TCR by peptide–MHC class II complex in conjunction with co-stimulatory signals. During T cell priming, cytokine receptor ligation can skew activated T cells into a particular effector cell type (6). These cell types are commonly referred to lineages or subsets, with each being identified by selected expression of characteristic transcription factors and effector molecules (7). With the exception of thymically derived regulatory T cells (Tregs) [reviewed in Ref. (8)], thymic emigrants lack any predisposition to make effector molecules associated with a particular CD4^+^ T cell subset, and require signals in the periphery to skew their differentiation into a particular cell subset. Present understanding of T cell lineage commitment is dominated by single fate model, a process whereby a naive T cell differentiates along a terminal fixed expression program, in response to signals at the time of antigen encounter. However, this view has needed revision in light of findings from many groups, which together demonstrate that CD4^+^ T cell subset fate is not a permanent attribute, but rather a flexible, plastic, feature that can be modified to suit the requirements of the immune system at a particular point in space and time. Thus, the new paradigm of T cell differentiation encompasses the ability of CD4^+^ T cells to change between expression programs traditionally thought to be mutually exclusive terminal states of differentiation (9). This feature has been reported *in vivo* in several experimental systems, and intriguingly, is a prominent feature of CD4^+^ T cell biology within the GALT.

The detailed mechanisms underlying T cell plasticity within the GALT remains to be defined, but several factors that facilitate its occurrence have been proposed and can be divided into extrinsic and intrinsic pathways. In this review, we will summarize the recent literature on CD4^+^ T cell plasticity in the gut, highlight possible underlying mechanisms and discuss its potential benefits for intestinal homeostasis and health.

### CD4^+^ T cell differentiation in the GALT

**Figure 1:**
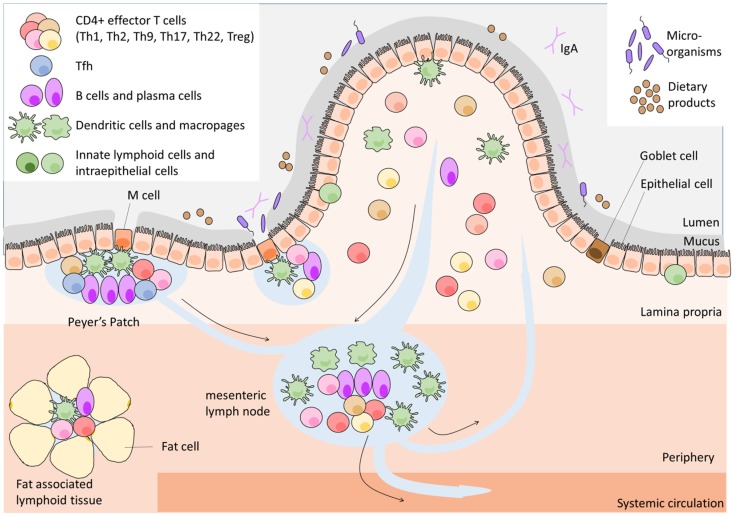
**Architecture of gut-associated lymphoid tissue**.

The GALT contains one of the largest lymphoid cell population found anywhere in the body. GALT is distributed along the intestinal tract and is separated from the luminal content, containing about 100 trillion microorganisms (10) and many dietary products, by a single epithelial layer covered with an intricate network of glycoproteins; the mucous layers. The GALT provides three functions: provides antigenic samples from throughout the GI tract; optimizes the opportunities for naïve lymphocytes to encounter antigen, and finally supports the activated lymphocyte and its initial differentiation. A network of highly organized lymphoid structures comprise the GALT (Figure 1) – including mesenteric lymph nodes (mLNs), Peyer’s patches (PPs), isolated lymphoid follicles (ILFs), cryptopatches (CPs), and fat-associated lymphoid tissues – as well as the loose connective tissue of the lamina propria (LP). Despite the numerous types of GALT, the organization of all GALT lymphoid tissues shares a basic cellular architecture that facilitates the interaction of APCs with lymphocytes and their subsequent activation and differentiation. However, the GALT tissues differ from each other both in function and in the physical distance to the intestinal lumen; CP and ILFs are located directly underneath the epithelial layer, PPs are further from the lumen and most distant are mLNs and fat-associated lymphoid tissues. The specialized functions of each the GALT tissues have been recently reviewed in Ref. (4), and therefore will not be discussed in detail in this review.

Naïve L-selectin-expressing CD4^+^ T cells migrate from the blood through high endothelial venules (HEVs) to the PPs, CP, ILFs, and mLNs. In these inductive sites of the adaptive immune system, they encounter their cognate antigen presented by APCs on MHC class II. Naïve T cells that did not encounter their cognate antigen leave via efferent lymphatic vessels into the systemic circulation to continue their search for their cognate antigen. In PPs, CP, and ILFs, dendritic cells (DCs) receive antigens transported from the mucosal surface by specialized epithelial cells overlying these lymphoid structures, the microfold (M) cells, and Goblet cells [reviewed in Ref. (4)]. mLNs receive antigens via afferent lymphatics that are connected with the PPs, while fat-associated lymphoid tissues obtain antigen by lymphatic drainage directly from the intestine and from the peritoneal cavity (4).

After activation in the intestine CD4^+^ T cells proliferate, shed L-selectin from their surface, increase surface expression of gut-specific adhesion molecules and differentiate into an effector T cell, of a particular subset. Antigen-experienced effector T cells usually leave via efferent lymphatic vessels and home to effector tissues such as the intestine after re-circulation in the blood, but some remain in the lymphoid organs to perform their specialized effector functions (11). Upon arriving in the intestine, effector T cells are likely to re-encounter their specific antigen presented by a diverse range of cells such as macrophages, B cells various types of DCs, and stromal and epithelial cells. This second antigen-specific interaction will trigger the T cells to execute their effector function.

It is the nature of antigen presentation and the surrounding micro-environment that largely seems to determine the outcome of CD4^+^ T cell differentiation and the subsequent spectrum of T cell identities leaving the lymphoid structures (6). The CD4^+^ T cell subsets currently recognized are: T helper cells of type-1 (Th1), Th2, Th9, Th17, Th22, Tregs, and follicular helper T cells (Tfh). Each subset is characterized by the potential to produce a defined set of mediators such as cytokines and chemokines, respond to particular stimuli via the expression of selected cytokine and chemokine receptors, largely driven by so called “master regulator” transcription factors (summarized in Table 1).

**Table 1:** **Effector CD4^+^ T cells in the gut**.

Cell type	Differentiation signals	Transcription factor (master transcription factor)	Cell surface and cytokine markers	Location in the gut under homeostatic conditions	Function in the gut
Th1	IL-12, IFNγ	*Tbet*, STAT1, STAT4, and Runx3	CXCR3, IFNγ	mLN, PP, CP, ILF, and LP	Responding to intracellular pathogens and assisting with viral infections
Th2	IL-4, IL-2	*GATA-3*, STAT6, and STAT5	IL-33R, IL-4, IL-13, and IL-5	Virtually absent	Responding to helminths
Th9	TGFβ, IL-4	PU.1	IL-9	Exact location to be determined	Involved in tumor immunology
Th17	IL-6, TGFβ, IL-1β, and (IL-23, IL-21)	*ROR*γ*t*, AhR, STAT3, Batf, Runx1, and RORα	CD161, IL-17A, IL-17F, and GM-CSF	mLN, PP, CP, ILF, and LP	Protect the host from infectious assault at mucosal site
Th22	IL-6, IL-13, and TNFα	AhR	CCR4, CCR6, CCR10, and IL-22	Exact location to be determined	Wound repair and induction of antimicrobials
Tfh	IL-6, IL-21	*Bcl-6*, Ascl2	CXCR5, PD-1, and IL-21	PP, ILF?	Induce IgA production by GC B cells to maintain a healthy microbiota
iTreg	TGFβ, IL-2	*Foxp3*, STAT5	IL-10, TGFβ	mLN, PP, CP, ILF, and LP	Suppressor capacity, keep homeostatic balance in gut

### CD4^+^ T cell plasticity

Plasticity of effector CD4^+^ T cells has been increasingly recognized in recent years. Initially, there were single reports of observations of particular subsets behaving “non-typically,” by the expression of transcription factors, cytokines, or both, of another subset. Together these various reports support a more nuanced view of Tfh differentiation, in place of the monolithic view of distinct, non-interchangeable lineages (12). T helper cells are able to acquire a mixed phenotype or to switch entirely to the transcription and cytokine profile of another lineage (Table 2). These hybrid cells or ex-Th lineage cells may either directly differentiate from naïve CD4^+^ T cells or arise during secondary immunological challenge. Current data suggest that, within the gut, particular transitions between CD4^+^ T helper cell subsets occur and are important for maintaining gastrointestinal homeostasis, as outlined below.

**Table 2:** **CD4^+^ T cell plasticity in the gut**.

Plasticity in the intestine	Capacity	Features of hybrid cell/ex-Th lineage cell	Origin	Developmental mechanism	Function in the gut	References
Th1/Th2	Th1/Th2 hybrid cells	Tbet^+^ CXCR3^+^ IFNg^+^ GATA-3^+^ IL-33R^+^ IL-4^+^ IL-13^+^ IL-15^+^	Naïve T cells	*H. polygyrus* infection →transcription factor balance	Gut function unknown; ↓ delayed-type hypersensitivity ↓allergic airway inflammation	(13)
Th17/Th1	Th17/Th1 hybrid cells	RORγt^+^ Tbet^+^ IL-17^+^ IFNγ^+^ CD161^+^ (only in humans) β7^+^ CCR6^+^	Naive T cells	Cytokine-induced upregulation of Tbet	Associated with Crohn’s disease	(14 –21)
	ex-Th17^Th1^ cells	Tbet^+^ RORγt^−^IL-17^−^IFNγ^+^ β7^+^ CCR6^+^	Committed Th17 cells	
Th17/Th2	Th17/Th2	RORγt^+^ GATA-3^+^ IL-4^+^ GM-CSF^+^ IL-17^+^	Not investigated	CD4-dependent Bcl11b-knock down or IL-4 treatment of EAE mice	↓EAE (b y redirecting lymphocytes to the gut)	(22)
Th17-to-Treg	Co-expression of Treg and Th17 markers not shown		Committed Th17 cells	PPARγ activation that promotes iTreg induction	↓T cell transferred colitis	(23)
Treg-to-Th17	Treg/Th17 hybrid cells	Foxp3^+^ RORγt^+^ IL-17^+^ β7^+^ CD103^+^ suppressive	Committed Treg cells	Micro-environmental cues in PPs of Crohn’s disease patients	↓Crohn’s disease	(24, 25)
Th17/Tfh	ex-Th17^Tfh^	RORγt^−^IL-17^−^Bcl-6^+^ CXCR5^+^ PD-1^+^ IL-21^+^	Committed Th17 cells	Not IL-23	Required for IgA-producing B cells	(26)
Treg/Tfh	ex-Treg^Tfh^ cells	Foxp3^−^Bcl-6^+^, IL-21^+^, CXCR5^+^, CD40L^+^, ICOS^+^, PD-1^+^, and CD28^+^	Committed Treg cells	Micro-environment of PPs. Requirement for B cells and CD40:CD40L interaction. Involvement of IL-6 and autocrine IL-21	Induced GC formation in PPs and IgA-producing cells in the gut	(27, 28)
CD4^+^/cytotoxic CD4^+^ T cells	CD4^+^ CTLs	CD4^+^ CD8αα^+^Runx3^+^ ThPOK^−^granzyme B^+^	CD4^+^ T cells	Continuous antigenic stimulation, IL-15, non-pathogenic microorganisms, TGFβ, RA	↓Colitis	(29, 30)

### Th1/Th2 cell plasticity

T helper cells of type-1 cells are an abundant effector population in the gut responsible for protection against invading intracellular bacteria and viruses (31). In contrast, Th2 cells are absent in the intestine of laboratory mice and humans under healthy conditions (31, 32); not surprising in view of their role in coordinating host responses to helminths and the absence of these organisms in a healthy intestinal flora (31). Identification of Th1 and Th2 as distinct cell types was the first, time where CD4^+^ T cell subsets were divided on the basis of phenotypic differences *in vitro* (33). It is therefore interesting that culture of T cells under mixed Th1 and Th2 conditions results in a continuum of cytokine expression, with some single positive IFNγ^+^ or IL-4^+^ and double positive cells, correlating with expression levels of Tbet and GATA-3 (34), the respective master transcription factors for the Th1 and Th2 subsets, demonstrating these cell fates are not a distinct as originally thought. Importantly, this finding is consistent with *in vivo* observations; during infections with *Heligmosomoides polygyrus*, a parasite that triggers a strong Th2 response, Th1/Th2 hybrid cells were observed (13). In the intestine, mLNs and spleen, these Th1/Th2 hybrid cells express simultaneously the Tbet and GATA-3 and not transcription factors of other subsets [retinoic acid (RA) receptor-related orphan nuclear receptor (ROR)γt or Bcl-6]. In addition, they express the Th1 cell-associated molecules CXCR3 and IFNγ concordantly with the Th2 cell-associated IL-33R, IL-4, IL-13, and IL-5. Furthermore, Th2 cells *in vivo* can express Tbet and IFNγ upon infection with lymphocytic choriomeningitis virus (LCMV), a potent Th1 skewing pathogen. These Th1/Th2 hybrid cells represent a stable phenotype, where attempts at reprograming by stimulation under Th1 or Th2 polarizing conditions result in quantitative changes of Th1/Th2 cytokine production without fully extinguishing either. Upon transfer into wild-type mice, around 20% of LCMV-specific Th1/Th2 cells maintained their expression of GATA-3, IL-4, and IL-13 alongside Tbet and IFNγ expression many months after systemic infection with LCMV. Earlier work from the same group has shown that the development of LCMV-specific Th1/Th2 hybrid cells originating from antigen-experienced Th2 cells required TCR engagement, not just a “Th1 milieu” provided by LCMV challenge and are stable *in vivo* for months (35).

Functionally, the Th1/Th2 hybrid cells have the capacity to elicit Th1 and Th2 cell responses but with a decreased Th-specific potency compared their single identity counterpart. Mice that received *in vitro* derived Th1/Th2 cells before the induction of type-1 inflammation showed signs for delayed-type hypersensitivity but not as strong as after a transfer of pure Th1 cells. Similarly, the transfer of Th1/Th2 cells reduced the signs for induced allergic airway inflammation compared to a Th2 cell transfer (13).

### Th17/Th1 cell plasticity

In health, Th17 cells preferentially home to small intestinal LP (26) and are important for intestinal homeostasis [reviewed in Ref. (36)]. Th17 cells are characterized by the expression of IL-17A, IL-17F, and IL-22 and the lineage-defining transcription factor RORγt (37–39), which acts in co-operation with other transcription factors, including aryl hydrocarbon receptor (AhR) and RORα (40, 41). Shortly after the first descriptions of Th17 cells, cells making both IL-17 and IFNγ, so called double producers, were noted. In the intestine of patients with Crohn’s disease (CD), IL-17A IFNγ-double positive CD4^+^ T cells co-expressing RORγt and Tbet have been found (14), sometimes with frequencies above those of single positive T cells (15). Cosmi et al. characterized these double producers further and found that virtually all IL-17^+^IFNγ^+^CD4^+^ T cells, as well as Th17 cells expressed the lectin-like surface molecule CD161 (15). This led them to conclude that human Th17 and Th17/Th1 cells exclusively originate from an NKT-like CD161^+^CD4^+^ T cell precursor. Furthermore, Kleinschek et al. showed that CD161^+^CD4^+^ T cells preferentially home to the gut due to their high expression levels of CCR6 and integrin β7. They confirmed that these cells can be induced to produce IFNγ in addition to IL-17 (16). In mice, colitis studies revealed not only the presence of Th1/Th17 hybrid cells (17–19) but also that Th17 cells, initially unable to produce IFNγ, abolish IL-17 production completely and switch on IFNγ (ex-Th17^Th1^) (18, 20, 42).

Hirota et al. used an IL-17-fate reporter mouse that permanently marks IL-17-producing cells to show that these ex-Th17^Th1^ cells undergo a near complete switch from Th17 cell to Th1 cell, except for the maintenance of IL-1R and AhR expression as remnants of their former identity (26). Since Th1 cells are not known for the expression of the IL-1R, ex-Th17^Th1^ cells are unique within the Th1 lineage in the ability to respond to the acute phase reactant IL-1. AhR expression has been shown to increase Th17 cell activity and particularly drive the expression of IL-22 (40). It is currently not known what the role of AhR is in ex-Th17^Th1^ cells, however, it is tempting to speculate that there is a role in coordinating IL-22 production at barrier sites such as the intestine, where IL-22R is expressed by epithelial cells (43). IL-22 deficiency worsens the colitis induced by either dextran sodium sulfate or by T cell transfer (44), and results in reduced secretion of antimicrobial agents important for maintaining epithelial health (45, 46). Interestingly, AhR has been shown essential for the long-term maintenance of lymphoid cells, such as innate lymphoid cells (ILC) type 3, intra-epithelial lymphocytes (IELs), and tissue resident cells, at epithelial barrier sites (47–49). This suggests that ex-Th17^Th1^ cells may be maintained in the intestine providing long-term protection or upholding aberrant immunity, in part through IL-22 signaling, with the help of AhR ligands.

Because the frequency and number of IL-17/IFNγ double producers are increased in the gut of CD patients (14), they have been implicated in disease pathogenesis. However, it is unknown if the presence of Th17/Th1 hybrid cells in CD patients are a cause or a consequence of the disease. These hybrid cells could develop as a bystander product of inflammation, or might drive the initial inflammatory response. In colitis models, the pathogenicity of Th1/Th17 hybrid cells and ex-Th17 cells is linked with Tbet. However, the role for Tbet in the pathogenicity of Th17 cells remains controversial (50–53) and switching to Th17/Th1 or IFNγ^+^ex-Th17 might involve Runx family members in co-operation with Tbet (54).

### Th17/Th2 cell plasticity

Naïve T cell activation *in vitro* in the presence of IL-4 inhibits RORγt expression and IL-17 production (55), which makes the existence of T cells with a hybrid phenotype of Th17/Th2 cells seem unlikely. However, *in vivo* studies identified CD4^+^ T cells simultaneously expressing IL-4 and IL-17 (22). These Th17/Th2 hybrid cells were reported to be present in draining LNs and mLNs of mice with experimental autoimmune encephalomyelitis (EAE) that either had a CD4-specific Bcl11b-deficency or were treated with IL-4. In EAE, Th17 cells are pathogenic when they gain access to the central nervous system (CNS), contributing to CNS inflammation. EAE-induced Bcl11b-deficient mice showed a delayed onset and reduced disease severity, attributed to a redirection of T helper cells away from a draining LN/CNS route to the intestine. Even though an accumulation of Th17 cells in the intestine has been reported to cause colitis these mice were asymptomatic.

### Th17-to-Treg cell plasticity

A different approach was chosen to address Th17 cell plasticity by Carbo et al. (23). Computational modeling predicated a role of the nuclear receptor peroxisome proliferator-activated receptor gamma (PPARγ) in a phenotype switch from Th17-to-Treg cell in the murine gut. *In silico*, PPARγ activation of fully differentiated Th17 cells reduced IL-17 and RORγt, while initiating Foxp3 expression. *In vitro*, analysis confirmed these *in silico* predictions. Polarized Th17 cells treated with pioglitazone, a synthetic PPARγ agonist, induced Foxp3 and inhibited RORγt and IL-17A expression. This effect was not observed in PPARγ-deficient Th17 cells. Mice that received pioglitazone orally to activate PPARγ recovered from transferred colitis, with a phenotypic switch in transferred T cells, from a predominantly Th17 phenotype to an iTreg cell phenotype characterized by decreased IL-17 and RORγt and increased expression of Foxp3.

In humans, IL-17^+^ FOXP3^+^ T cells were identified in inflamed intestinal mucosa of patients with CD, but not in patients with ulcerative colitis or healthy controls (24). These Treg/Th17 cells shared characteristics of Th17 and Treg cells, expressing *RORC* (encoding RORγt) and CD161 and showing Treg-typical suppressor activity *in vitro*. In addition, the majority these FOXP3^+^IL-17^+^ T cells expressed CCR6, a receptor that mediates homing to skin and mucosal tissues (56) and high levels of integrin α4β7 and CD103, markers for gut-homing potential. Analysis of the TCR repertoire suggested that FOXP3^+^IL-17^+^ cells develop from FOXP3^+^ Tregs when exposed to inflammatory signals in the gut (24) and *in vitro* stimulation of FOXP3^+^ circulating cells can result in IL-17 expression (57). In human tonsils obtained after routine tonsillectomy, up to 25% of the FOXP3^+^CD4^+^ T cell population can produce IL-17 upon activation (25). These FOXP3^+^IL-17 producers are CCR6^+^, co-express *RORC* and *FOXP3* and display cell-contact dependent suppressive properties (25). Whilst FOXP3^+^IL-17^+^ cells in the LP appear unique to areas of intestinal inflammation in CD (24), in tonsil they are much more readily identifiable (25). Together these observations suggest that heterogeneity in the GALT micro-environment can influence Th17–Treg plasticity. Intriguingly, the FOXP3^+^IL-17^+^ T cells from CD patients also express IFNγ, IL-22, and IL-21 (24), suggesting plasticity not just between Th17 and Treg, but with other CD4^+^ effector states.

### Th17/Tfh cell plasticity

In addition to a Th17-to-Th1 conversion, Th17 cells can acquire a Tfh cell phenotype (26), including expression of Bcl-6, the transcription factor that is both necessary and sufficient for the formation of Tfh (58–60). Tfh provide antigen-specific help to germinal center (GC) B cells via CD40L, IL-4, and IL-21 (61). The only GALT to contain GCs are the PP and mLNs, and within these structures Tfh act to select high-affinity GC B cells to exit the PP GC as long-lived IgA secreting plasma cells and memory B cells (62). An IL-17-fate reporter mouse was used to show that transfer of Th17 cells into a T cell deficient recipient could support high-affinity IgA production in the GCs of PPs by converting into Tfh cells (26). Interestingly, this conversion resulted in the loss of IL-17 expression and other Th17 associated features to enable these cells to effectively switch into Tfh cells in the PP. The physiological relevance of these ex-Th17^Tfh^ cells in host defense or in microbial homeostasis remains to be elucidated. But, the essential role for Th17 cells in the production of high-affinity IgA (26) suggests that this Th17 to Tfh cell switch is critical for IgA-dependent immune responses.

IgA production in the gut can occur outside the GC but the IgA species are low-affinity, with little somatic hypermutation (63). The production of GC-derived IgA is essential for the maintenance of bacterial communities in the gut. Mice that express a mutated form of activation-induced cytidine deaminase (AIDG23S) that allows class switch to IgA but not somatic hypermutation have alterations of the gut microbiota, suggesting that IgA derived from the PP GC response is essential for maintaining homeostasis of the gut microbiota (64). This demonstrates that high-affinity IgA promotes healthy microbiota, rather than target it for elimination like IgG derived from the LN and spleen (62). The production of immunoglobulin that nourishes commensal microbiota is one of the features that discriminates the function of GALT GCs from peripheral GCs, and may be one of the reasons that GALT GC T cells exhibit plasticity that is not observed in the GCs of lymph nodes and spleen. Selective IgA deficiency (OMIM 137100) is a common incidental finding amongst blood donors (with numerous reports since the 1970s), without any particular symptomatology originally appreciated [reviewed in Ref. (65)]. However, it has recently been reported that IgA-deficient persons have increased frequency of gastrointestinal and respiratory infections, and of allergy and autoimmunity (66).

### Treg-to-Tfh cell plasticity

In mice, Th17 cells are not unique in their ability to switch to a Tfh cell phenotype, also Foxp3^+^ cells have been reported to be able to do this in the PP GCs (27, 28). The first study used adoptive transfer of CD4^+^EGFP^+^ cells from spleen or LNs of Foxp3–EGFP reporter mice into T cell deficient mice (27). Whilst 80% of Foxp3^+^ T cells switched off Foxp3 expression in the PPs, about half of the transferred cells kept their Foxp3^+^ profile in the LP of the small intestine as well as in the spleen. The ex-Treg^Tfh^ cells that form in the PP expressed Bcl-6 and also IL-21, CXCR5, CD40L, ICOS, PD-1, and CD28, all associated with Tfh cell function (61). Functionally, it has been shown that these ex-Treg^Tfh^ cells induce GC formation in PPs and facilitate IgA-producing cells in the gut (27). These findings appear in contrast with the work of Hirota et al. that identified ex-Th17^Tfh^ cells (26). In the study by Hirota et al., mucosal Foxp3^+^ T cells transferred into TCRα^−/−^ hosts did not differentiate into Tfh cells, induce GC B cells or IgA production. It remains unclear whether differences in the models (Tsuji: CD3ε^−/−^ recipients; Hirota: TCRα^−/−^ recipients) or source of cells transferred (Tsuji: spleen and LN, Hirota: mucosal origin) or intestinal environment of the transfer recipients explain the discrepancies. In a separate study, Takahashi et al. transferred iTreg cells into TCRα^−/−^ mice and showed that these cells can lose Foxp3 expression and become Tfh in the PP (67). Intriguingly, transferred iTreg cells were more likely to lose Foxp3 expression in the PP than in the spleen, suggesting that the micro-environment of the gut affects the plasticity of Tregs. The conversion of Treg-to-Tfh in the gut is controlled, in part, by expression of microRNA-10a that suppresses Bcl-6 and Ncor2, thereby repressing of Treg-to-Tfh conversion in the gut (67).

Not all Tregs within the PP convert into Tfh, however, there is a regulatory population of CD4^+^ T cells in GCs; T follicular regulatory cells (Tfr) (68–70). Tfr are the progeny of Foxp3^+^CD4^+^ cells that share phenotypic characteristics of Tfh cells, including CXCR5, PD-1, and Bcl-6 expression. In peripheral secondary lymphoid organs, Tfr control the size and output of the GC response (68–70). In PP GCs, Tfr control the quality of IgA produced, which in turn promotes diversity within the microbial community of the gut (28). Taken together, conversion of Treg-to-Tfh in the gut is finely balanced to ensure the ratio of Tfh to Tfr within the PP supports high-affinity IgA production and homeostasis of commensal bacteria.

### Th2-to-Tfh cell plasticity

The gastrointestinal helminth parasite *H. polygyrus* has been shown to give raise to Th1/Th2 hybrid cells but also been suggested give rise to Th2/Tfh hybrid cells (71). These hybrids were described as IL-4 producing CD4^+^ cells expressing CXCR5, ICOS and PD-1, Bcl-6 and IL-21 and that localized in B cell follicles of mLNs after *H. polygyrus* infection. Zaretsky et al. found similar IL-4^+^CXCR5^+^PD-1^+^ Th cells after immunization with schistosomiasis soluble egg antigen (72). A later study using dual reporters for IL-4 and IL-13 and *N. brasiliensis* infection demonstrated that Tfh phenotype cells express IL-4 but not IL-13, whereas Th2 cells express both prototypic Th2 cytokines. Importantly, IL-4^+^ Tfh cells are Bcl-6^+^ and do not express high levels of GATA-3, suggesting that these cells are Tfh cells that produce IL-4 rather than *bona fide* Th2/Tfh hybrids (73).

### CD4^+^ T cell into cytotoxic CD4^+^ T cell conversion

Among the CD4^+^ IELs a population of cells has been described that lost its CD4-lineage transcription factor ThPOK and acquired the CD8-lineage transcription factor Runx3 (29, 30). The induction of Runx3 expression by CD4^+^ T cells was associated with expression of CD8αα, the natural killer cell – and CTL-related molecule 2B4 (CD244) and Tbet (29). In addition, these cells expressed granzyme and the activation-induced degranulation marker CD107a (LAMP-1) (30). As well as closely resembling mature CD8^+^ CTLs, these cells showed high cytolytic activity. At the same time, these cells lose their ability to produce IL-17 and to express RORγt (29, 30). Fate-mapping and adoptive transfer experiments by Mucida et al. showed that CD4^+^ CTLs originate from ThPOK^+^ naive CD4^+^ T cells (30).

The loss of ThPOK together with the inability to produce IL-17 in the intestine had physiological implications on the development of colitis. Conditional deletion of ThPOK in CD4^+^ T cells prior to their transfer into lymphopenic Rag^−/−^ deficient mice enhanced the differentiation of CD4^+^CD8α^+^ CTLs in the gut of the recipients, which, as a consequence, saw a reduction in intestinal inflammation compared with controls (29). By contrast, the transfer of Runx3-deficient CD4^+^ T cells resulted in an exacerbated intestinal inflammation, but provided recipient mice with increased protection against infection with the enteropathogenic bacterium *Citrobacter rodentium* (29). A role of CD4^+^ CTL cells in celiac disease is suggested by their restriction to MHC class II, their induction by dietary antigen, responsiveness to IL-15, and cytotoxic potential (30).

### Mechanisms of CD4^+^ T cell plasticity

Very little is known about the underlying mechanisms determining CD4^+^ T cell plasticity. It is likely that the same factors that are involved in T cell differentiation are implicated in a phenotype switch, but this remains largely untested.

**Figure 2:**
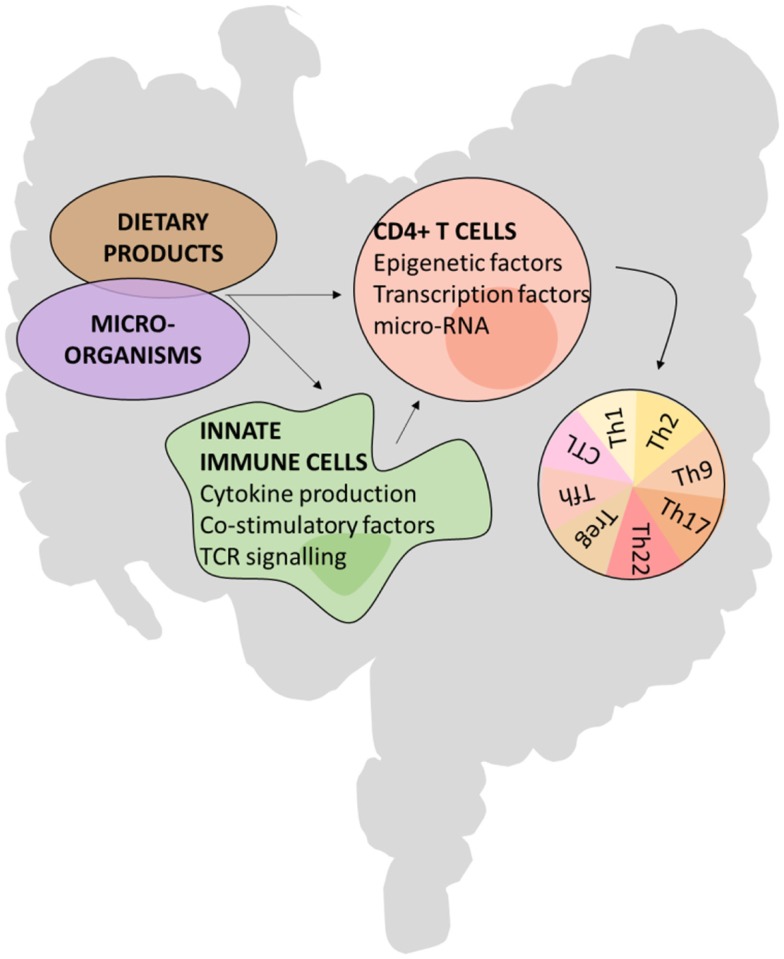
**Possible mechanisms of CD4^+^ T cell plasticity in the gut**.

The development of some CD4^+^ T hybrid cells or ex-Th lineage cells seem to predominantly take place in the intestine, indicating that the micro-environment has a major influence on CD4^+^ T cell plasticity and identity. For example, the micro-environmental cues contributing to a phenotype switch between Th17 and Tfh seem to be unique to the PP, as the plasticity of Th17 cells toward a Tfh cell fate was restricted to the micro-environment of PPs and did not occur in peripheral LNs (26). Likewise, transferred Foxp3^+^ T cells lost Foxp3 expression and acquired Tfh features preferentially in the PPs (27, 65). Further evidence of the unique nature of the PP comes from *in vitro* stimulation assays in which APCs from PP and spleen generate different CD4^+^ effectors (74, 75). Factors enabling CD4^+^ T cell plasticity could, in part, originate from the microbiota or dietary products that effect CD4^+^ T cells either directly or indirectly via innate immune cells. These extrinsic cues can alter transcription factor and microRNA expression and epigenetic markers such as histone modifications (Figure 2). For example, microRNA-10a dampens the conversion of Treg cells to both Th17 and Tfh cell fates (67). In addition, the cellular composition of the GALT, enriched in non-conventional lymphocytes such as ILCs, IEL populations of TCRγδ^+^, and CD8αα^+^TCRαβ^+^ T cells and mucosal associated innate NKT cells, is substantially different compared with other secondary lymphoid organs and will contribute to a different environment in which T cells encounter antigen and are maintained.

### Microbiota

The nature of antigen or metabolic products provided by the gut microbiota plays a role in T cell plasticity. There are a number of examples where CD4^+^ T cell subsets are expanded in response to particular microbial species and their products. Using germ-free mice, a mixture of 17 *Clostridia* species were selected from human gut flora for their ability to induce mucosal Tregs (76). Introduction of these 17 strains mitigated colitis and allergic diarrhea models (76). This protective effect is mediated by short chain fatty acids, products of these 17 bacteria (and of *Bacteroides fragilis*, also shown to induce Tregs) (77). The microbiome is shaped, in part by induced Tregs, and mice lacking induced Tregs are more susceptible to colitis and asthma (78). Thus the gut microbiome is implicated in the protection of autoimmunity and autoinflammation both in the gut and at distant sites. Recent work provides evidence for a symbiosis between CD4^+^ T cell subsets and the microbiome (28). Lack of Tfr resulted in poor quality IgA production and a limited biodiversity of the microbiome. The presence of Tfr permitted a more diverse microbiome including a larger representation of non-pathogenic *Clostridia* (28), which establishes a positive feedback loop between CD4^+^ cells and the microbiome mediated through SCFA, Foxp3 expression, and IgA production. This narrowing of the microbiome and subsequently SCFA deplete environment might explain the association between IgA deficiency and allergy and autoimmunity (66). Taken together, these studies provide evidence that the gut microbiota and CD4^+^ T cell fate within the gut are inter-dependent, with each affecting the composition of the other.

CD4^+^CD8α^+^ CTLs are absent from the intestines of germ-free mice and of mice mono-colonized with segmented filamentous bacteria (SFB), but appear in the intestines following reconstitution with specific non-pathogenic microorganisms (30). Little is known about the mechanisms how antigens originating from microbiota induce a phenotype switch. However, data on Th17 cell differentiation indicate that SFB colonization promotes Th17 cell commitment by the expression of inflammation-associated genes, such as the gene for serum amyloid A (SAA) (79). Some Th17 cells from murine gut have TCR specific for SFB-derived antigen (80). In addition, it has been shown that ATP derived from commensal bacteria activates CD70^high^ CD11c^low^ cells in the LP, leading to enhanced differentiation of Th17 cells (81). Therefore, changes in the concentration of SAA or ATP could either induce a switch toward Th17 phenotype or destabilize Th17 cell commitment toward another phenotype.

### Dietary products

Dietary products, mainly after being processed by microorganisms, can induce phenotype switch. PPARγ has been identified to induce a Th17-to-Treg cell switch (23). This nuclear receptor regulates fatty acid storage and glucose metabolism. Therefore, consumption of food containing certain fatty acids may promote a phenotype switch from Th17-to-Treg cells. Several dietary products have been reported to promote or inhibit lineage commitment [reviewed in Ref. (2)]. It is likely that these factors are also candidates to induce a phenotype switch of CD4^+^ T cells. In addition, dietary products could influence a phenotype switch by affecting metabolic and signaling pathways and epigenetic status.

### Innate immune cells: Providers of costimulation and cytokines

Innate immune cells are abundant in the GALT. They have the potential to influence CD4^+^ T cell plasticity via their determination of the micro-environment via the secretion of soluble mediators, expression of co-stimulatory molecules, and via their potential to act as APCs. Mucida et al. demonstrated that continuous activation of CD4^+^ T cells by oral administration of an antigen is necessary for a phenotype switch to CD4^+^ CTLs (30). It has been reported previously that antigen dose and peptide/TCR affinity can influence Th commitment (82).

Co-stimulatory factors have been shown to play a role in Treg-to-Tfh plasticity. Blocking the interaction of CD40, expressed on APCs such as B cells and DCs, with CD40L, found on T cells, is able to prevent a phenotype switch from Treg cell to Tfh cell (27).

The role of cytokines in Th17 cell plasticity has been studied by several investigators but has not yet been fully elucidated. However, it is clear that IL-23-dependent pathway does play an important role. In *Helicobacter hepaticus*-induced typhlocolitis, mRNA levels for IL-23p19 were elevated after bacterial inoculation and *ex vivo* IL-17A^+^ cells isolated from the colitic intestine expressed both subunits of the IL-23R, indicating that IL-23 acts on Th17 cells to induce a program resulting in IFNγ production (18). T cell transfer studies also showed that IL-23 is required for the appearance of IL-17A^+^IFNγ^+^ double-producing T cells in the intestine. Transferred naive CD4^+^ T cells developed to Th1/Th17 hybrid cells in Rag^−/−^ mice, but not in p19^−/−^ RAG^−/−^ mice [lacking IL-23; (21)]. These studies were extended by Ahern et al. demonstrating that in the intestine the emergence of IL-17A^+^IFNγ^+^ double-producing Th cells, but not IL-17A single producing Th cells, requires T cell-intrinsic IL-23 signaling by transferring IL-23R^−/−^CD45RB^high^ CD4^+^ T cells into full hosts (17). In humans, CD161^+^CD4^+^ T cells from CD patients readily produce IL-17 and IFNγ upon stimulation with IL-23, whereas, in healthy subjects, IL-1β was required alongside IL-23 (16).

The role of IL-12, the primary cytokine inducing Th1 cell differentiation, in mediating Th17-to-Th1 cell conversion remains controversial. *In vitro*, obtained results differ from those data being observed in *in vivo* models. Upon transfer of naive CD4^+^ T cells, Th17/Th1 cells were detected in the mLNs of IL12p35-Rag-double deficient mice (lacking IL-12), demonstrating that IL-12 is negligible in Th1/Th17 induction in the intestine (21). In contrast, Lee et al. showed *in vitro* that in both IL-23 and IL-12 are able to switch off IL-17 and enhance IFNγ production in a STAT4- and Tbet-dependent manner. It has also been shown that *ex vivo* Th17 cells can be converted into a Th1-like phenotype following IFNγ-induced expression of Tbet and acquisition of IL-12Rβ2 surface expression (83, 84). Lee et al. investigated the effect of additional cytokines on Th17 cell plasticity and found that Th17 cells require TGFβ for sustained expression of IL-17F and IL-17A (20).

Although the precise role of IL-23 in the Th17-to-Th1 cell conversion requires further investigation, it does not seem to play an essential role in a phenotype switch from Th17 to Tfh cell. Th17 cells can give rise to Tfh cells in IL-23-competent and -deficient mice (26). For a phenotype switch from Tregs to Tfh cells it has been shown that the cytokine IL-6 can to down-regulate Foxp3, and this may be relevant to Treg plasticity in the IL-6 rich PPs (85).

### Transcription factors

Transcription factors maintain and instruct lineage programs by complex mechanisms, often involving several cooperating transcription factors, and their binding and access to specific DNA sequences. The expression of lineage determining transcription factors is induced by multiple factors in the micro-environment, including cytokines. Their regulation falls outside the scope of this review, but their interactions are of importance in maintaining Th identity and are likely to play a prominent role in ultimately generating hybrid Th cells and Th identity conversions. Co-expression of IFNγ and Th2-associated cytokines in the same T cell seems to be enabled by signals that keep both Tbet and GATA-3 expression in balance, which is in contrast to studies examining chromatin modification at *Tbx21* and *Gata3* (86, 87).

The loss of a single transcription factor can tip the balance in favor of an alternative lineage. In Bcl11b-deficient mice, GATA-3 expression in Th17 cells is unrestrained, resulting in GATA-3-mediated IL-4 production (22). This change in cell phenotype feeds back to the micro-environment, and has further implications for T cell biology. For example, the cytokine mix produced by Th2/Th17 hybrid cells triggers gut-imprinting properties in DCs. IL-4 together with GM-CSF enhance the expression of the enzyme RALDH2 in DCs (88, 89), leading to elevated levels of RA (90) that imprint gut-homing properties on T cells, such as α4β7 and CCR9 (91). Similarly, the termination of ThPOK in mature CD4^+^ T cells enables the de-repression of the CTL program (30), resulting in MHC class II restricted CD4^+^ T cells with a cytotoxic effector function. This process can be further promoted by Runx3 (29).

The factors responsible for carefully balancing two transcriptional programs within a single T cell may be present together from the moment of T cell differentiation or may be induced later. *In vitro*, data have shown that expression of one differentiation program is often found to be mutually exclusive with factors from another (55). Yet, differentiation of some programs are characterized by co-expression of two pathways from the start, such as seen with iTreg differentiation in which T cells co-express Foxp3 and RORγt from the start (25). These *in vitro* observations are often not recapitulated *in vivo* and vice versa. This highlights missing links in our understanding of which factors are required to determine full Th effector cell differentiation, which factors enable T cells to postpone the identity decision, as well as those that can still interfere at later stages.

### Epigenetics

The observed stability or plasticity of Th subsets is governed by the epigenetic regulation of the key transcription factors and cytokines and their access to downstream genes (92). Whether a particular gene is poised for expression is controlled by the chromatin structure and histone or DNA modification state. Wei et al. evaluated the histone H3 methylation status over the entire genome in a variety of defined Th subsets (87). Promoter regions for *Tbx21* (encoding Tbet) in Th1 cells and *Rorc* (encoding RORγt) in Th17 cells displayed a permissive methylation state (H3K4me3) associated with full expression of these master regulators in each lineage. However, the promoter region for *Tbx21* in Th17 cells had a bivalent status characterized by H3K4me3/H3K27me3, reflecting the relative instability of these cells and their potential to acquire another Th cell phenotype. In addition, it has been shown that treatment of naïve T cells under Treg polarizing conditions with the fatty acid, butyrate, enhances permissive acetylation in the promoter region of the *Foxp3* locus. Butyrate, being a large bowel fermentation product, provides a link between microbial products and Treg cells by epigenetic mechanisms. Beyond histone modifications, miRNAs offer a further tuning of gene expression and CD4^+^ phenotype, as illustrated earlier.

## Function

CD4^+^ T cell plasticity is important in the resolution of infections but can contribute to immunopathogenesis. The ability to switch or combine effector phenotypes can generally be seen as a very useful feature in the arsenal of the adaptive immune system. It is an additional tool providing flexibility to adequately control rapidly changing microorganisms. It allows the retention of TCRs with a useful track record while displaying a high degree of flexibility and adaptation depending on local environmental cues. Therefore, a phenotype switch may facilitate the ability to respond more quickly to changing immunological challenges and to successfully terminate immune responses after pathogen clearance. This flexibility may be especially important and useful at epithelial interfaces, where luminal content is constantly changing and the largest population of microorganism is encountered.

The ability to switch from one phenotype to another or obtain features from two effector programs increases the efficiency of a response. Combining effector mechanisms would enable the control of complex organisms that have evolved ways to deal with one predominant effector subset. This implies that effector programs can co-exists within one T cell, while the co-existences of different effector subset within the same micro-environment may be harder to achieve and maintain. It is worth noting that reports on Th cells displaying a hybrid phenotype highlight that such cells are less effective in dealing with situations where one part of their identity is required compared with the single identity T cells. This indicates that each identity is weakened by the presence of the transcriptional program of the other, a feature that could be abused by microorganisms. Conversely, producing these “weakened” T cells may be method of peripheral tolerance – in an inflamed milieu, T cells are stimulated under polarizing conditions and develop a single identity, whereas non-inflamed milieu offers conditions less favorable to any single identity and the “weakened” T cells are tolerized.

An additional physiological role for CD4^+^ T cell plasticity is the gaining of specific functional features that require aspects of the previous full identity combined with a very specific trade. This is the case for Tfh cells that can develop from committed Th17 or Treg cells. In case of ex-Th17^Tfh^ cells, they represent a subset of Tfh cells that are specialized to promote high-affinity IgA production in the GCs of PPs. A similar specialized role in GC B cell support is likely the case for ex-Treg^Tfh^. These Tfh adopters have only been found in the PP and only support IgA production, demonstrating that this plasticity is unique to the gut environment.

Lastly, the transcriptional programs of each T cell effector subset may not be compatible with long-term survival. Effector cells that switch to a Th1 cell phenotype, may be maintained better as memory cells due to the activity mediated by Tbet. Recent reports describe a role of Tbet expression gradient as a regulator of CD4^+^ T cell memory formation, with highly Th1-polarized Tbet^+^ cells displaying end-effector features and a short lifespan, whereas Th cells with a lower expression of Tbet are able to enter the long-lived memory T cell pool (93, 94). This is to some degree mimicked by the finding that long-lived ex-Th17 cells acquired Tbet expression, albeit at levels lower than their Th1 counterparts.

From a therapeutic viewpoint, contemplating a deliberately induced phenotype switch could be used to up- or down-regulate the different effector arms of the adaptive immune response. Dampening CD4^+^ T cell function would be useful for autoimmune disorders including CD and multiple sclerosis. In a colitis model, oral pioglitazone induces a switch from Th17 to an iTreg phenotype, improving disease and in EAE (a model of MS) pathogenic Th17 cells can be redirected to gut, again improving disease. Enhanced CD4^+^ T cell responses could improve cancer surveillance and clearance. Whilst phenotypic switching appears to occur in very specific circumstances, some of these conditions are encountered spontaneously in healthy animals. Thus strategies to induce phenotype switching, with minimal side effects or co-morbidities, might be identifiable.

## Conclusion

The nuanced view of Th differentiation suggested that within any CD4^+^ effector population there are varying ratios of lineage determining transcription factors in each cell, with correspondingly graded cellular phenotypes. In GALT these ratios integrate a number of different signals – microbial products, dietary influences, costimulation, cytokines, and epigenetic modifications. Variation in these signals and their interpretation is the substrate for heterogeneity of CD4^+^ T cells. As these microbial and dietary cues are unique to GALT, perhaps even unique to the most luminal GALT, such as PP, they could together provide a micro-environment permissive for plasticity, in contrast to other secondary lymphoid organs, where plasticity is less frequently reported and where much of the rigid *in vivo* definitions of the CD4^+^ subsets were derived. Whether the phenotypic switches described herein are unique to an *in entero* environment is unknown. It is possible that similar blurring of CD4^+^ subsets occurs at other mucosal sites, balancing tolerance, protection, and immunopathology with the need to maintain a diverse microbiome. To understand the precise factors underlying plasticity is relevant for our understanding of host (and microbiomial) health, host defense, pathogenesis of various gastrointestinal diseases and also for development and optimization of vaccines, where an enforced Tfh phenotype is desired.

## Conflict of Interest Statement

The authors declare that the research was conducted in the absence of any commercial or financial relationships that could be construed as a potential conflict of interest.
